# Investigation of the Effects of Artemisinin on Acetaminophen-Induced Acute Liver Toxicity

**DOI:** 10.1021/acs.chemrestox.6c00158

**Published:** 2026-06-10

**Authors:** Pelin Kurt, Rustem Anil Ugan, Aylin Aydin, Nevra Aydemir Celep, Elif Cadirci

**Affiliations:** † Department of Medical Pharmacology, Faculty of Medicine, 37503 Atatürk University, Erzurum 25240, Türkiye; ‡ Department of Pharmacology, Faculty of Pharmacy, 37503 Atatürk University, Erzurum 25240, Türkiye; § Department of Veterinary Histology and Embryology, Faculty of Veterinary Medicine, 37503 Atatürk University, Erzurum 25240, Türkiye

## Abstract

Paracetamol (PARA) is a widely used antipyretic and analgesic drug; however, overdose is a major cause of hepatotoxicity. Although *N*-acetylcysteine (NAC) is the standard antidote, its limitations necessitate the investigation of alternative therapeutic approaches. Artemisinin (ART), a natural compound with antioxidant and anti-inflammatory properties, was evaluated for its hepatoprotective potential in a paracetamol-induced acute liver injury model. Biochemical liver function parameters (ALT, AST), oxidative stress markers (SOD, GSH, MDA), molecular markers (TNF-α, IL-1β, iNOS, NF-κB, CYP2E1 mRNA expression), and histopathological changes were assessed using spectrophotometric assays, quantitative real-time PCR (qPCR), and histological staining methods. PARA administration significantly increased ALT and AST levels compared to the CONTROL group (*p* < 0.001). Among the treatment groups, PARA + ART 14 significantly reduced these enzyme levels compared to the PARA group (*p* < 0.001). ART treatment also increased antioxidant parameters (SOD, GSH) and decreased MDA levels compared to the PARA group (*p* < 0.001), with effects comparable to PARA + NAC. Inflammation- and oxidative stress-related gene expressions were significantly elevated in the PARA group versus CONTROL (*p* < 0.001), whereas PARA + ART 14 significantly downregulated all markers (*p* < 0.001). Histopathological findings supported the biochemical and molecular results, showing partial improvement in PARA + ART 7 and more pronounced recovery in PARA + ART 14, while no significant improvement was observed in PARA + ART 35. These findings suggest that ART may exert dose-dependent hepatoprotective effects against paracetamol-induced liver injury by modulating oxidative stress and inflammatory responses. Further studies are needed to determine the optimal therapeutic dose and confirm its clinical potential.

## Graphical abstract

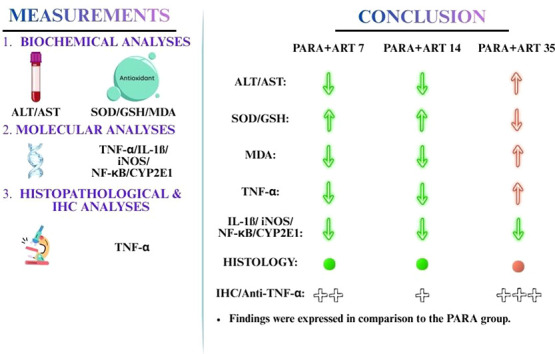

## 1 Introduction

Acetaminophen, also known as paracetamol (*N*-acetyl-*p*-aminophenol, PARA), was first introduced for clinical use in 1955.^1^ Today, it is a widely used drug with antipyretic and analgesic properties.^2^

Following oral administration, PARA is rapidly absorbed from the stomach and small intestine and is metabolized in the liver.^3^ Peak serum concentrations are reached 1.5–2 h after ingestion. The therapeutic serum concentrations of PARA have been reported as 10–20 μg/mL (65–130 μmol/L).^4^

In children, the therapeutic dose is 10–15 mg/kg, with a recommended maximum daily dose of 50–90 mg/kg. In adults, the therapeutic dose is 325–1000 mg every 4–6 h, with a recommended maximum daily dose of up to 4 g.^3^ A single oral dose of ≥250 mg/kg, or ingestion of more than 12 g within 24 h, carries a high risk of liver toxicity.^3b,5^ Severe hepatotoxicity is typically observed at doses exceeding 350 mg/kg.^6^

At therapeutic doses, approximately 90% of PARA is metabolized in the liver through conjugation with sulfate and glucuronide, and the resulting metabolites are excreted in the urine.^7^ Of the remaining 10%, about 2% is excreted unchanged in the urine, while roughly 8% is converted by hepatic cytochrome P450 enzymes (CYP2E1, CYP1A2, CYP3A4) into *N*-acetyl-*p*-benzoquinone imine (NAPQI), a highly reactive and hepatotoxic compound.^8^ As illustrated in Figure 1, inhibition of CYP2E1 activity, which plays a key role in this pathway, may reduce the severity of PARA-induced hepatotoxicity.^9^

**Figure 1:**
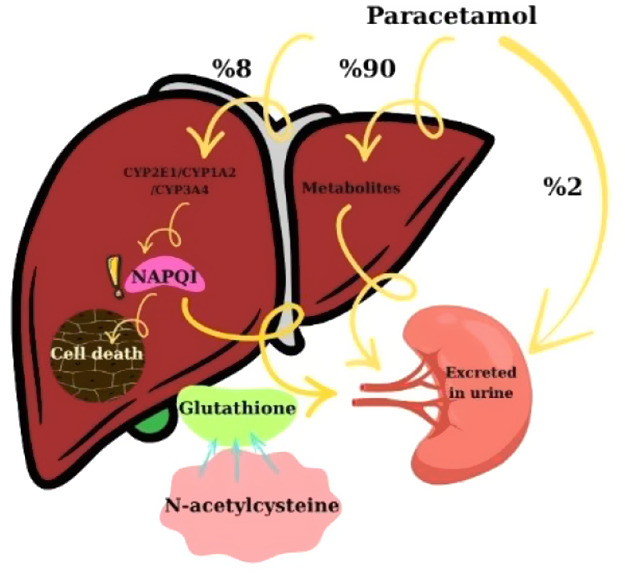
Schematic representation of paracetamol (PARA) metabolism in the liver.

Under normal conditions, hepatic glutathione (GSH) detoxifies the toxic metabolite NAPQI by converting it into nontoxic metabolites. However, in situations leading to GSH depletion (such as PARA overdose, chronic alcoholism, or malnutrition), NAPQI cannot be detoxified and inevitably causes liver injury.^10^

*N*-Acetylcysteine (NAC) is traditionally used as an antidote to prevent liver injury. NAC, a GSH precursor, was first introduced as an antidote in the United Kingdom in the 1970s.^11^ By increasing depleted hepatic GSH levels, NAC prevents PARA poisoning. Administration within the first 10 h after PARA ingestion largely prevents the development of liver damage.^12^

Artemisinin (ART) is the major compound of *Artemisia absinthium*, commonly known as wormwood. Literature reports indicate that ART possesses a wide range of pharmacological effects, including anticancer, anti-inflammatory, and antioxidant activities.^13^ In PARA toxicity, ART is considered to provide potential additional benefits not only through its antioxidant and GSH precursor effects, but also by inhibiting endothelial damage, inflammation, and angiogenesis occurring during hepatotoxicity.^13^

Based on this rationale, the present study aimed to investigate the potential of ART as an adjunct to NAC therapy in preventing paracetamol-induced liver injury. Although previous studies have demonstrated the hepatoprotective effects of *Artemisia* species extracts as well as ART, the effects of ART on paracetamol-induced acute liver toxicity have not yet been sufficiently elucidated. Therefore, this study was designed to address this gap in the literature and to further clarify the potential hepatoprotective role of ART in this model.

## 2 Materials and Methods

### 2.1 Experimental Animals

In this study, a total of 35 male Albino Wistar rats, weighing between 200 and 220 g, were obtained from the experimental animal laboratory of the Experimental Research and Application Center (ATADEM) at Atatürk University. During the experimental period, the rats were provided with water and pellet feed ad libitum. Prior to the experiment, the animals were housed in groups under standard laboratory conditions at a normal room temperature of 22 °C and fed accordingly. All procedures of the present study were conducted in accordance with ethical guidelines and were approved by the Atatürk University Local Ethics Committee for Animal Experiments (AÜHADYEK) in the meeting dated October 26, 2023 (No: 2023/12) and the official letter dated November 1, 2023 (No: 2300352321).

Artemisinin (ART) was purchased from Sigma-Aldrich (St. Louis, MO, USA) with a reported purity of ≥98%.

### 2.2 Formation of Experimental Groups

The rats in the study were randomly divided into seven groups, each consisting of five animals. All groups were fasted for 24 h prior to the experiment.

The fasted animals were subjected to the experimental protocols outlined below:

**Group I (CONTROL):** 2 mL distilled water administered orally via gavage.
**Group II (ART 35):** 35 mg/kg ART administered orally via gavage.
**Group III (PARA):** 2 g/kg PARA suspension administered orally via gavage.
**Group IV (PARA + NAC):** 140 mg/kg NAC administered orally, followed by 2 g/kg PARA suspension administered orally via gavage after 1 h.
**Group V (PARA + ART 7):** 7 mg/kg ART administered orally, followed by 2 g/kg PARA suspension administered orally via gavage after 1 h.
**Group VI (PARA + ART 14):** 14 mg/kg ART administered orally, followed by 2 g/kg PARA suspension administered orally via gavage after 1 h.
**Group VII (PARA + ART 35):** 35 mg/kg ART administered orally, followed by 2 g/kg PARA suspension administered orally via gavage after 1 h.

All PARA doses administered in the study were determined based on the relevant literature.^14^ The doses of ART (7, 14, and 35 mg/kg) were selected based on previous studies reporting its hepatoprotective and antioxidant effects, and to evaluate potential dose-dependent responses.^15,16^ Immediately after PARA administration, all rats were provided with water and pellet feed ad libitum until the end of the experiment.

24 h after PARA administration, anesthesia was induced in all groups by intraperitoneal (i.p.) injection of 5 mg/kg xylazine and 75 mg/kg ketamine, after which the experiment was terminated. Blood samples were collected from all animals, and their livers were harvested. A portion of each liver was allocated for biochemical and molecular analyses and stored at −80 °C. The remaining portion was fixed in 10% formalin solution for histopathological examination. Serum samples were stored at −80 °C.

Prior to biochemical and molecular analyses, the tissues of each animal were individually homogenized in liquid nitrogen using a mortar and pestle, followed by homogenization in stainless steel jars with a TissueLyser II (Qiagen, Hilden, Germany).

### 2.3 Biochemical Analyses

#### 2.3.1 Serum ALT and AST Measurements

Measurements were performed at the Veterinary Diagnosis and Analysis Laboratory of the Faculty of Veterinary Medicine, Atatürk University, using the Monaco/Randox X Auto-Chemistry Analyzer device.

#### 2.3.2 Measurement of SOD, GSH, and MDA in Liver Tissue

A 100 mg sample from each tissue was homogenized in 1 mL PBS buffer using the Tissue Lyserr device. After centrifugation, the supernatant was used for biochemical analyses. Superoxide dismutase (SOD) activity,^17^ GSH^18^ and malondialdehyde (MDA) levels^19^ were determined using modified methods based on our previous studies and measured spectrophotometrically with a Multiscan Sky Microplate (Thermo) device.^20^ Protein concentrations were determined manually by the Lowry method. The mean absorbance of each sample and standard was calculated. All data were expressed as the mean ± standard deviation (SD) per mg protein.

### 2.4 Molecular Analyses

#### 2.4.1 Measurement of TNF-α, IL-1β, iNOS, NF-κB, and CYP2E1 mRNA Expression

In this study, TNF-α, IL-1β, iNOS, NF-κB, and CYP2E1 mRNA expression levels were compared among the groups.

#### 2.4.2 RNA Isolation from the Rat Liver Tissue

Liver tissues were homogenized using the Tissue Lyser II device (by pooling within groups, with 350 μL RLT buffer added to 20 mg tissue) and RNA extraction was performed with the QIAcube RNA isolation system (Qiagen, Hilden, Germany). Each tissue sample was weighed individually, and total RNA isolation was carried out using the RNeasy Mini Kit (Qiagen) according to the manufacturer’s protocol. The concentration and purity of total mRNA were determined by nanodrop spectrophotometry (Multiscan Sky Microplate, Thermo).

#### 2.4.3 Reverse Transcription Reaction and cDNA Synthesis

cDNA was synthesized from total RNA using the High-Capacity cDNA Reverse Transcription Kit (Applied Biosystems). Each reaction was performed with 10 μL of RNA. cDNA synthesis was conducted according to the specified thermal conditions using the Veriti 96 Well Thermal Cycler (Applied Biosystems). The cDNA concentration was determined by nanodrop spectrophotometry (Multiscan Sky Microplate, Thermo).

#### 2.4.4 Quantitative Determination of mRNA Expression by Real-Time PCR

The mRNA expression levels of TNF-α, IL-1β, iNOS, NF-κB, and CYP2E1 were quantified using the TaqMan Gene Expression Master Mix (Applied Biosystems). Amplification and quantification were performed on the StepOne Plus Real-Time PCR System (Applied Biosystems, CA, USA). For 200 ng of cDNA, TaqMan Gene Expression Assays specific for TNF-α (Rn01525859), IL-1β (Rn00580432), iNOS (Rn00561646), NF-κB (Rn01399581), and CYP2E1 (Rn00580624) (Thermo Scientific) were used, and 40 cycles were run. The Ct values were automatically converted to delta–delta Ct by the instrument, and all data were analyzed using the 2^–ΔΔCt^ method, with results expressed as fold change relative to the CONTROL group.^21^

### 2.5 Histopathological Analyses

#### 2.5.1 Light Microscopic Procedures

All conventional light microscopy procedures were performed at the Laboratory of the Department of Histology and Embryology, Faculty of Veterinary Medicine, Atatürk University.

Liver tissues obtained from all experimental groups were fixed in 10% formaldehyde solution for 72 h. Following fixation, tissues were washed in running water, dehydrated through a graded alcohol series (70, 80, 96, and 100%), cleared in xylene, and embedded in paraffin. Sections of 5 μm thickness were obtained using a Leica RM2125RT microtome.

Sections were deparaffinized in xylene and rehydrated through graded alcohols. Nuclei were stained with hematoxylin for 1 min, followed by washing with distilled water and incubation in sodium thiosulfate solution for 2 min. Cytoplasmic staining was performed with acid fuchsin, and connective tissue was stained with aniline blue solution. Sections were dehydrated, cleared in xylene, and mounted with Entellan.

#### 2.5.2 Immunohistochemical Staining Procedure

Tissue sections on adhesive slides were deparaffinized and rehydrated. Endogenous peroxidase activity was blocked with 3% hydrogen peroxide for 10 min. Antigen retrieval was performed in citrate buffer solution (pH 6.0) at 500 W for 15 min in a microwave oven. Nonspecific binding was blocked with protein block for 10 min.

Sections were incubated with TNF-α Monoclonal Antibody (Q34; dilution: 1/100; BT-MCA1210) at +4 °C for 24 h. Following primary antibody incubation, complement (Thermo Fisher, DAB Substrate System) was applied for 30 min and conjugate for 25 min, with PBS washing at each step. Visualization was performed using DAB (3,3′-diaminobenzidine) chromogen, and counterstaining was carried out with Mayer’s hematoxylin. Slides were dehydrated, cleared in xylene, and mounted with Entellan.

#### 2.5.3 Microscopic Imaging and Photography

Microscopic imaging was performed using a Zeiss AXIO Scope.A1 microscope equipped with a camera attachment. Image processing was performed using Adobe Photoshop CS6.

Histopathological evaluations were performed by two independent investigators blinded to the treatment groups. A minimum of three paraffin sections were obtained from each animal, and at least 10 high-power fields (HPF) were systematically examined at 20× magnification per section. Histopathological lesions were evaluated using a semiquantitative scoring system; necrosis, vacuolization, sinusoidal dilatation, inflammation, and fatty change were each scored on a scale of 0–3 (0 = absent, 1 = mild, 2 = moderate, 3 = severe). Statistical comparisons between groups were performed using the Kruskal–Wallis test, followed by Bonferroni-corrected Mann–Whitney *U* pairwise comparisons. All data are expressed as mean ± standard deviation (SD). A *p* value of <0.05 was considered statistically significant.

### 2.6 Statistical Analyses

The results obtained from the experiments were expressed as mean ± standard deviation, and *p* values below 0.05 were considered statistically significant. The significance of differences between groups was determined using one-way analysis of variance (ANOVA) followed by the Tukey post hoc test, performed with the IBM SPSS 20 software.

Asterisks in the columns (*p < 0.05, **p < 0.01, and ***p < 0.001) indicate statistically significant differences between the CONTROL group and other groups according to the Tukey test. Sigma symbols (^σ^ *p* < 0.05, ^σσ^ *p* < 0.01, and ^σσσ^ *p* < 0.001) indicate statistically significant differences between the PARA group and the protective treatment groups according to the Tukey test. Groups without symbols showed no significant differences.

## 3 Results

### 3.1 Biochemical Findings

#### 3.1.1 Effects of Artemisinin on Serum ALT and AST Levels

ALT and AST levels were significantly increased in the PARA group (180.29 ± 13.00 and 240.91 ± 18.39 U/L, respectively) compared to the CONTROL group (41.79 ± 3.40 and 67.60 ± 4.51 U/L, respectively) (*p* < 0.001) (Figure 2).

**Figure 2:**
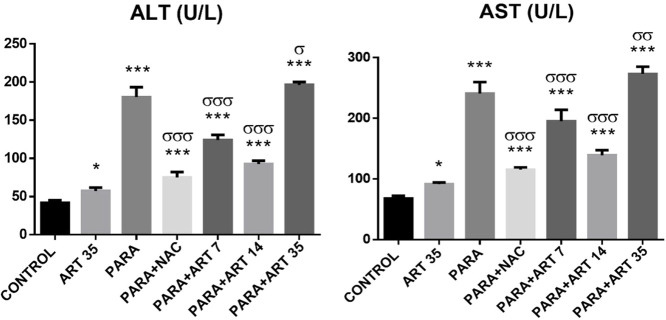
Effects of artemisinin on serum ALT and AST levels in CONTROL, ART 35, PARA, PARA + NAC, PARA + ART 7, PARA + ART 14, and PARA + ART 35 groups. Data are presented as mean ± SD (*n* = 5/group). Results are presented as mean ± standard deviation. Asterisks * (*p* < 0.05) and *** (*p* < 0.001) indicate statistically significant differences compared to the CONTROL group, as determined by the Tukey test. Symbols σ (*p* < 0.05), σσ (*p* < 0.01), and σσσ (*p* < 0.001) indicate statistically significant differences compared to the PARA group, as determined by the Tukey test.

Treatment with NAC and ART significantly reduced ALT and AST levels compared to the PARA group. Specifically, ALT and AST levels in the PARA + NAC group (75.19 ± 6.94 and 115.40 ± 3.65 U/L, respectively), PARA + ART 7 group (124.00 ± 6.60 and 195.20 ± 18.29 U/L, respectively), and PARA + ART 14 group (92.60 ± 4.39 and 139.07 ± 8.09 U/L, respectively) were significantly lower than those in the PARA group (*p* < 0.001 for all comparisons) (Figure 2).

A dose-dependent improvement was observed between the ART-treated groups, with the PARA + ART 14 group demonstrating a more pronounced reduction compared to the PARA + ART 7 group.

A dose-dependent reduction was observed between the results of the PARA + ART 7 and PARA + ART 14 groups. In contrast, treatment with ART at 35 mg/kg resulted in a significant increase in ALT and AST levels (196.40 ± 3.65 and 273.00 ± 12.04 U/L, respectively) compared to the PARA group (180.29 ± 13.00 and 240.91 ± 18.39 U/L, respectively), with statistical significance observed for ALT (*p* < 0.05) and AST (*p* < 0.01) (Figure 2).

### 3.2 Effects of Artemisinin on Hepatic SOD, GSH, and MDA Levels

SOD activity and GSH levels were significantly decreased in the PARA group (13.80 ± 0.84 U/mg protein and 1.44 ± 0.05 nmol/mg protein, respectively) compared to the CONTROL group (51.60 ± 5.94 U/mg protein and 4.14 ± 0.26 nmol/mg protein, respectively) (*p* < 0.001) (Figure 3). MDA levels were significantly increased in the PARA group (4.22 ± 0.08 nmol/mg protein) compared to the CONTROL group (1.70 ± 0.07 nmol/mg protein) (*p* < 0.001) (Figure 3). Compared to the PARA group, the PARA + NAC group showed significant improvement in all parameters, including increased SOD activity (37.80 ± 3.35 U/mg protein) and GSH levels (3.34 ± 0.11 nmol/mg protein), along with decreased MDA levels (2.20 ± 0.07 nmol/mg protein) (*p* < 0.001 for all) (Figure 3).

**Figure 3:**
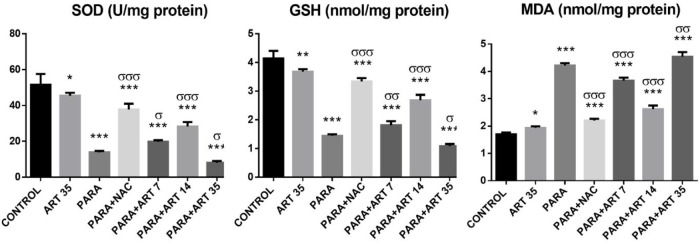
Effects of artemisinin on mean SOD Activity, GSH and MDA levels in liver tissue of CONTROL, ART 35, PARA, PARA + NAC, PARA + ART 7, PARA + ART 14 and PARA + ART 35 groups. Data are presented as mean ± SD (*n* = 5/group). Results are presented as mean ± standard deviation. The symbols * (*p* < 0.05), ** (*p* < 0.01), and *** (*p* < 0.001) indicate statistically significant differences compared to the CONTROL group, as determined by the Tukey test. The symbols σ (*p* < 0.05), σσ (*p* < 0.01), and σσσ (*p* < 0.001) indicate statistically significant differences compared to the PARA group, as determined by the Tukey test.

In the PARA + ART 7 group, SOD activity (19.80 ± 0.84 U/mg protein) (*p* < 0.05), GSH levels (1.81 ± 0.14 nmol/mg protein) (*p* < 0.01), and MDA levels (3.66 ± 0.11 nmol/mg protein) (*p* < 0.001) were significantly improved compared to the PARA group (Figure 3).

In the PARA + ART 14 group, all parameters, including SOD activity (28.20 ± 2.49 U/mg protein), GSH levels (2.68 ± 0.19 nmol/mg protein), and MDA levels (2.62 ± 0.13 nmol/mg protein), showed significant improvement compared to the PARA group (*p* < 0.001 for all) (Figure 3).

A dose-dependent improvement was observed among ART-treated groups, with a more pronounced effect in the PARA + ART 14 group compared to the PARA + ART 7 group.

In contrast, in the PARA + ART 35 group, SOD (8.00 ± 1.00 U/mg protein) and GSH (1.08 ± 0.07 nmol/mg protein) levels were significantly decreased compared to the PARA group (SOD: 13.80 ± 0.84 U/mg protein, GSH: 1.44 ± 0.05 nmol/mg protein) (*p* < 0.05), while MDA levels (4.54 ± 0.17 nmol/mg protein) were significantly increased compared to the PARA group (4.22 ± 0.08 nmol/mg protein) (*p* < 0.01) (Figure 3).

### 3.3 Molecular Findings

#### 3.3.1 Effects of Artemisinin on TNF-α, IL-1β, iNOS, and NF-κB mRNA Levels in the Liver Tissue

**TNF-α:** TNF-α mRNA expression levels were determined as 1.01 ± 0.04 fold in the CONTROL group, while an increase to 1.30 ± 0.10 fold was observed in the ART 35 group. In the PARA group, TNF-α mRNA expression was significantly elevated to 5.52 ± 0.17 fold compared to the CONTROL group (*p* < 0.001) (Figure 4).

**Figure 4:**
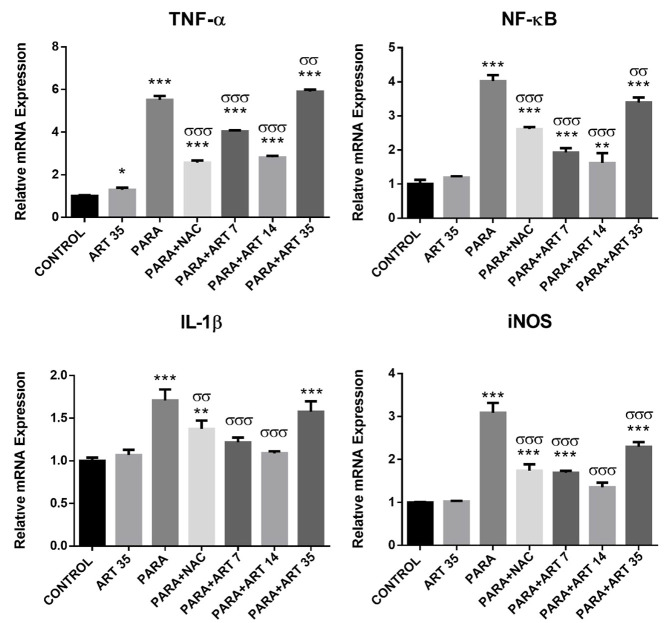
Effects of artemisinin on TNF-α, IL-1β, iNOS, and NF-κB mRNA levels in liver tissue of CONTROL, ART 35, PARA, PARA + NAC, PARA + ART 7, PARA + ART 14, and PARA + ART 35 groups. Gene expression levels were analyzed by qPCR using the 2^–ΔΔCt^ method (*n* = 5/group). Results are presented as mean ± standard deviation. Columns marked with * (*p* < 0.05), ** (*p* < 0.01), and *** (*p* < 0.001) indicate statistically significant differences compared to the CONTROL group, as determined by Tukey test. Groups without such markings show no significant differences. Columns marked with σσ (*p* < 0.01) and σσσ (*p* < 0.001) indicate statistically significant differences compared to the PARA group, as determined by Tukey’s test. Groups without these markings show no significant differences.

Compared to the PARA group, TNF-α mRNA expression was significantly reduced to 2.57 ± 0.10 fold in the PARA + NAC group (*p* < 0.001), decreased to 4.04 ± 0.05 fold in the PARA + ART 7 group (*p* < 0.001), and further reduced to 2.82 ± 0.07 fold in the PARA + ART 14 group (*p* < 0.001). Notably, the reduction observed in the PARA + ART 14 group was more pronounced than that in the PARA + ART 7 group, indicating a dose-dependent effect (Figure 4).

In contrast, TNF-α mRNA expression in the PARA + ART 35 group increased to 5.9 ± 0.10 fold, showing a significant elevation compared to the PARA group (*p* < 0.01). These findings suggest that while ART exhibits anti-inflammatory effects at low and moderate doses, this effect may be lost at higher doses, potentially leading to an exacerbation of the inflammatory response (Figure 4).

**IL-1β:** IL-1β mRNA expression levels were determined as 1.00 ± 0.037 fold in the CONTROL group, while a slight increase to 1.068 ± 0.061 fold was observed in the ART 35 group. In the PARA group, IL-1β mRNA expression was significantly elevated to 1.71 ± 0.129 fold compared to the CONTROL group (*p* < 0.001) (Figure 4).

Compared to the PARA group, IL-1β mRNA expression was significantly reduced to 1.376 ± 0.097 fold in the PARA + NAC group (*p* < 0.01), decreased to 1.217 ± 0.057 fold in the PARA + ART 7 group (*p* < 0.001), and further reduced to 1.089 ± 0.024 fold in the PARA + ART 14 group (*p* < 0.001). Notably, the reduction observed in the PARA + ART 14 group was more pronounced than that in the PARA + ART 7 group, indicating a dose-dependent effect (Figure 4).

In contrast, IL-1β mRNA expression in the PARA + ART 35 group increased to 1.577 ± 0.122 fold; however, this increase was not statistically significant compared to the PARA group (*p* > 0.05). These findings suggest that while ART exhibits anti-inflammatory effects at low and moderate doses, this effect may be diminished at higher doses (Figure 4).

**iNOS:** iNOS mRNA expression levels were determined as 1.00 ± 0.009 fold in the CONTROL group, while a slight increase to 1.023 ± 0.017 fold was observed in the ART 35 group. In the PARA group, iNOS mRNA expression was significantly elevated to 3.088 ± 0.257 fold compared to the CONTROL group (*p* < 0.001) (Figure 4).

Compared to the PARA group, iNOS mRNA expression was significantly reduced to 1.743 ± 0.163 fold in the PARA + NAC group (*p* < 0.001), decreased to 1.690 ± 0.052 fold in the PARA + ART 7 group (*p* < 0.001), and further reduced to 1.354 ± 0.120 fold in the PARA + ART 14 group (*p* < 0.001). Notably, the reduction observed in the PARA + ART 14 group was more pronounced than that in the PARA + ART 7 group, indicating a dose-dependent effect (Figure 4).

In contrast, iNOS mRNA expression in the PARA + ART 35 group increased to 2.300 ± 0.116 fold, but remained significantly lower than the PARA group (*p* < 0.001). However, the relatively elevated levels compared to other treatment groups suggest a diminished anti-inflammatory effect at higher doses (Figure 4).

**NF-κB:** NF-κB mRNA expression levels were determined as 1.005 ± 0.123 fold in the CONTROL group, while a slight increase to 1.195 ± 0.036 fold was observed in the ART 35 group. In the PARA group, NF-κB mRNA expression was significantly elevated to 4.023 ± 0.175 fold compared to the CONTROL group (*p* < 0.001) (Figure 4).

Compared to the PARA group, NF-κB mRNA expression was significantly reduced to 2.614 ± 0.060 fold in the PARA + NAC group (*p* < 0.001), decreased to 1.927 ± 0.129 fold in the PARA + ART 7 group (*p* < 0.001), and further reduced to 1.620 ± 0.293 fold in the PARA + ART 14 group (*p* < 0.001). Notably, the reduction observed in the PARA + ART 14 group was more pronounced than that in the PARA + ART 7 group, indicating a dose-dependent effect (Figure 4).

In contrast, NF-κB mRNA expression in the PARA + ART 35 group increased to 3.396 ± 0.149 fold, but remained significantly lower than the PARA group (*p* < 0.01). However, the relatively elevated levels compared to other treatment groups suggest a diminished anti-inflammatory effect at higher doses (Figure 4).

#### 3.3.2 Effects of Artemisinin on CYP2E1 mRNA Levels in Liver Tissue

CYP2E1 mRNA expression levels were determined as 1.001 ± 0.044 fold in the CONTROL group, while a slight increase to 1.033 ± 0.039 fold was observed in the ART 35 group. In the PARA group, CYP2E1 mRNA expression was significantly elevated to 1.890 ± 0.071 fold compared to the CONTROL group (*p* < 0.001) (Figure 5).

**Figure 5:**
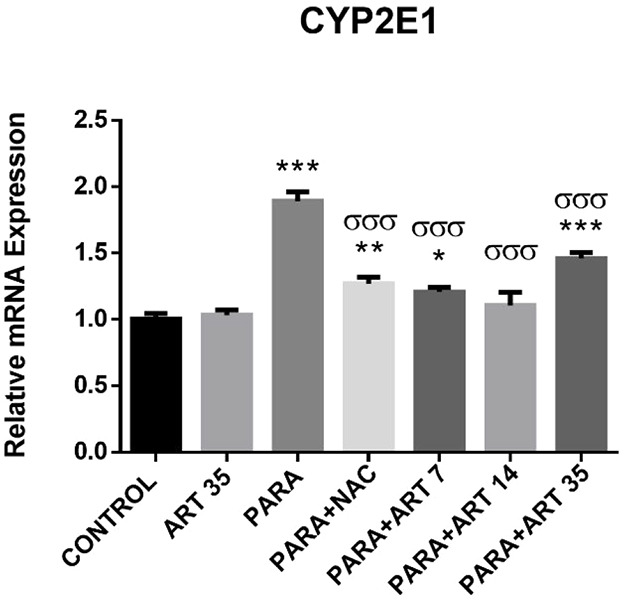
Effects of artemisinin on CYP2E1 mRNA levels in liver tissue of CONTROL, ART 35, PARA, PARA + NAC, PARA + ART 7, PARA + ART 14 and PARA + ART 35 groups. Gene expression levels were analyzed by qPCR using the 2^–ΔΔCt^ method (*n* = 5/group). Results are presented as mean ± standard deviation. Columns marked with * (*p* < 0.05), ** (*p* < 0.01), and *** (*p* < 0.001) indicate statistically significant differences compared to the CONTROL group, as determined by the Tukey test. Groups without symbols show no significant differences. Columns marked with σσσ (*p* < 0.001) indicate statistically significant differences compared to the PARA group and the protective treatment groups, as determined by the Tukey test.

Compared to the PARA group, CYP2E1 mRNA expression was significantly reduced to 1.270 ± 0.050 fold in the PARA + NAC group (*p* < 0.001), decreased to 1.208 ± 0.034 fold in the PARA + ART 7 group (*p* < 0.001), and further reduced to 1.105 ± 0.099 fold in the PARA + ART 14 group (*p* < 0.001). Notably, the reduction observed in the PARA + ART 14 group was more pronounced than that in the PARA + ART 7 group, indicating a dose-dependent effect (Figure 5).

In contrast, CYP2E1 mRNA expression in the PARA + ART 35 group increased to 1.459 ± 0.046 fold, but remained significantly lower than the PARA group (*p* < 0.001). However, the relatively elevated levels compared to other treatment groups suggest a diminished hepatoprotective effect at higher doses (Figure 5).

### 3.4 Histopathological Findings

Kruskal–Wallis analysis of histopathological scores revealed a statistically significant difference among groups (*H* = 29.121, *p* < 0.001). Pairwise comparisons were performed using Bonferroni-corrected Mann–Whitney *U* test.

Histopathological examination of liver sections from the control group revealed normal histological architecture, with hexagonally shaped lobules, portal triads at the periphery, a central vein at the center, and hepatocytes arranged radially in normal hepatic cord configuration. The total histopathological score of the control group was 1.60 ± 0.55, representing the lowest value among all groups (Figure 6).

**Figure 6:**
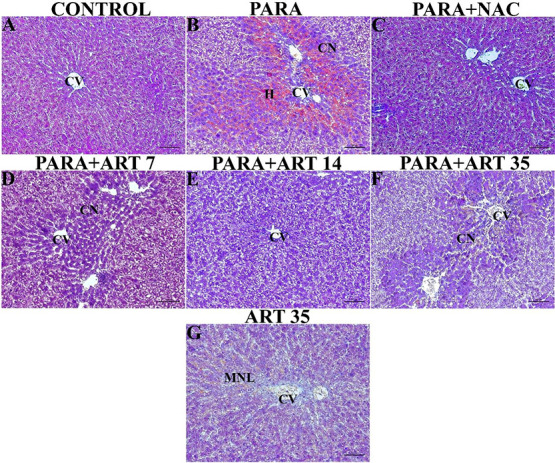
Photomicrographs of liver tissues from control, paracetamol-treated, and artemisinin-treated rats at different doses. (A) Control group: normal lobular architecture with portal triads and radial hepatocyte arrangement around the central vein. (B) Paracetamol group: severe centrilobular necrosis, sinusoidal dilatation, disruption of hepatocyte membrane integrity, and hemorrhagic foci. (C) Paracetamol + NAC group: markedly reduced necrotic areas and hepatocyte damage, with near-normal lobular architecture. (D) Paracetamol + artemisinin 7 mg/kg group: persistent necrotic areas and hepatocyte damage with no evident recovery. (E) Paracetamol + artemisinin 14 mg/kg group: marked reduction in necrotic areas and sinusoidal dilatation, with improvement in lobular architecture. (F) Paracetamol + artemisinin 35 mg/kg group: persistent centrilobular necrosis, sinusoidal dilatation, and hemorrhagic foci. (G) Artemisinin 35 mg/kg alone group: prominent necrotic cells and mononuclear cell infiltration. Mallory’s triple stain, modified by Crosman; objective lens: 20×; scale bar: 50 μm. CV: central vein; CN: centrilobular necrosis; H: hemorrhagic areas; MNL: mononuclear cell infiltration.

In the paracetamol-treated group, severe hepatocyte vacuolization, sinusoidal dilatation, portal tract inflammation, fatty changes, and necrosis were detected. Disruption of hepatocyte membrane integrity and the presence of hemorrhagic foci were prominent findings. The total histopathological score was 13.60 ± 0.55, which was significantly higher than that of the control group (*p* = 0.0097) (Figure 6).

In the paracetamol + *N*-acetylcysteine (NAC) group, a marked reduction in tissue damage was observed, with evident recovery of normal lobular architecture. The total histopathological score was 7.60 ± 2.41, which was significantly lower than that of the paracetamol group (*p* = 0.0109) (Figure 6).

In the paracetamol + artemisinin 7 mg/kg group, hepatocyte damage and necrotic areas persisted prominently, with a total histopathological score of 12.80 ± 1.30. No statistically significant difference was detected compared with the paracetamol group (*p* = 0.3661) (Figure 6).

In the paracetamol + artemisinin 14 mg/kg group, tissue damage was markedly reduced, with a notable decrease in necrotic areas and sinusoidal dilatation compared with the higher-dose groups. The total histopathological score was 8.40 ± 1.34, which was significantly lower than both the paracetamol group (*p* = 0.0086) and the paracetamol + artemisinin 7 mg/kg group (*p* = 0.0095) (Figure 6).

In the paracetamol + artemisinin 35 mg/kg group, centrilobular necrosis, sinusoidal dilatation, and hepatocyte damage persisted at high levels, with a total histopathological score of 12.00 ± 1.41. No statistically significant difference was detected compared with either the paracetamol group (*p* = 0.0969) or the paracetamol + artemisinin 7 mg/kg group (*p* = 0.3808) (Figure 6).

In the artemisinin 35 mg/kg alone group, prominent necrotic cells and mononuclear cell infiltration were observed. The total histopathological score was 11.40 ± 0.55, which was significantly higher than that of the control group (*p* = 0.0097) (Figure 6). Histopathological scoring results for all groups and parameters are summarized in Table 1.

**Table 1:** Histopathological Scoring Results in Liver Tissue (Mean ± SD)^a^

group	necrosis	vacuolization	sinusoidal dilatation	inflammation	fatty change	total score
CONTROL	0.40 ± 0.55	0.20 ± 0.45	0.20 ± 0.45	0.40 ± 0.55	0.40 ± 0.55	1.60 ± 0.55
ART 35	2.80 ± 0.45	2.80 ± 0.45	2.60 ± 0.55	2.80 ± 0.45	2.60 ± 0.55	13.60 ± 0.55
PARA	1.60 ± 0.55	1.40 ± 0.55	1.60 ± 0.55	1.80 ± 0.84	1.20 ± 0.45	7.60 ± 2.41
PARA + NAC	2.60 ± 0.55	2.60 ± 0.55	2.60 ± 0.55	2.60 ± 0.55	2.40 ± 0.55	12.80 ± 1.30
PARA + ART 7	1.60 ± 0.55	1.80 ± 0.84	1.60 ± 0.55	2.00 ± 0.71	1.40 ± 0.55	8.40 ± 1.34
PARA + ART 14	2.20 ± 0.45	2.80 ± 0.45	2.20 ± 0.45	2.60 ± 0.55	2.20 ± 0.45	12.00 ± 1.41
PARA + ART 35	2.60 ± 0.55	2.20 ± 0.45	2.40 ± 0.55	2.40 ± 0.55	1.80 ± 0.45	11.40 ± 0.55

a Score: 0 = absent, 1 = mild, 2 = moderate, 3 = severe. Values are presented as mean ± SD (*n* = 5/group).

### 3.5 Immunohistochemical Findings

Kruskal–Wallis analysis of TNF-α immunoreactivity scores revealed a statistically significant difference among groups (*H* = 25.071, *p* < 0.001). Pairwise comparisons were performed using a Bonferroni-corrected Mann–Whitney *U* test.

In the control group, TNF-α expression was minimal (0.20 ± 0.45) and no prominent immunopositivity was observed. In the paracetamol group, TNF-α expression was markedly elevated (2.60 ± 0.55), with intense immunopositivity particularly around portal areas and the central vein; this score was significantly higher than that of the control group (*p* = 0.0086). In the paracetamol + NAC group, TNF-α immunoreactivity (1.40 ± 0.55) was significantly lower than that of the paracetamol group (*p* = 0.0269). In the paracetamol + artemisinin 7 mg/kg group, TNF-α immunoreactivity (2.60 ± 0.55) remained elevated and did not differ significantly from the paracetamol group (*p* = 1.0000). In the paracetamol + artemisinin 14 mg/kg group, TNF-α expression (1.40 ± 0.55) was significantly lower than both the paracetamol group (*p* = 0.0269) and the paracetamol + artemisinin 7 mg/kg group (*p* = 0.0269). In the paracetamol + artemisinin 35 mg/kg group, TNF-α expression (2.80 ± 0.45) remained elevated and did not differ significantly from the paracetamol group (*p* = 0.6005). In the artemisinin 35 mg/kg alone group, TNF-α immunoreactivity (2.60 ± 0.55) was significantly higher than that of the control group (*p* = 0.0086). TNF-α immunostaining findings for all groups are presented in Figure 7, and anti-TNF-α immunoreactivity scores are summarized in Table 2.

**Table 2:** Anti-TNF-α Immunoreactivity Scores in Liver Tissue (Mean ± SD)^a^

group	anti-TNF-α (mean ± SD)	immunoreactivity
CONTROL	0.20 ± 0.45	negative (−)
ART 35	2.60 ± 0.55	strong positive (+++)
PARA	2.60 ± 0.55	strong positive (+++)
PARA + NAC	1.40 ± 0.55	weak positive (+)
PARA + ART 7	2.60 ± 0.55	moderate positive (++)
PARA + ART 14	1.40 ± 0.55	weak-moderate positive (+/++)
PARA + ART 35	2.80 ± 0.45	strong positive (+++)

a Score: 0 = negative, 1 = weak positive (+), 2 = moderate positive (++), 3 = strong positive (+++). Values are presented as mean ± SD (*n* = 5/group). Kruskal–Wallis: *H* = 25.071, *p* < 0.001.

**Figure 7:**
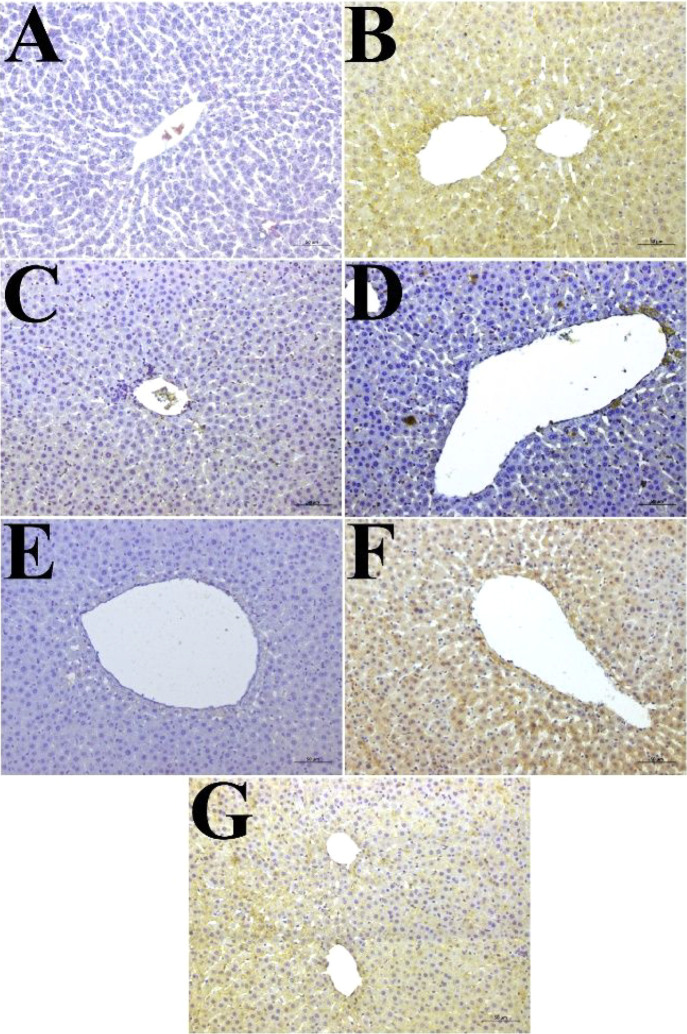
Photomicrographs of liver tissue immunohistochemically stained with anti-TNF-α. (A) Control group: minimal TNF-α expression with no prominent immunopositivity. (B) Paracetamol group: intense TNF-α immunopositivity, particularly around portal areas and the central vein. (C) Paracetamol + NAC group: markedly reduced TNF-α expression with suppression of the inflammatory response. (D) Paracetamol + artemisinin 7 mg/kg group: persistently elevated TNF-α immunoreactivity with prominent inflammatory cell infiltration in portal areas. (E) Paracetamol + artemisinin 14 mg/kg group: reduced TNF-α expression with attenuation of immunoreactivity in hepatocytes. (F) Paracetamol + artemisinin 35 mg/kg group: intense TNF-α immunopositivity persisting in centrilobular regions. (G) Artemisinin 35 mg/kg alone group: widespread immunopositivity in portal areas and hepatocytes with continued inflammatory response. Avidin–biotin method; DAB chromogen; Mayer’s hematoxylin counterstain; magnification: 200×.

## 4 Discussion

In today’s world, PARA is a widely used and easily accessible analgesic and antipyretic drug.^2^ When administered at therapeutic doses, PARA possesses a reasonable safety profile; however, when taken in supratherapeutic doses, whether intentionally or accidentally, it can lead to severe hepatotoxicity or serious conditions such as liver failure.^22^

In PARA overdose, the glucuronidation and sulfation pathways become saturated, and the normal hepatic GSH levels are insufficient to inactivate the toxic NAPQI metabolite. In cases of PARA poisoning, the traditionally recognized antidote NAC exerts its effect by increasing hepatic GSH levels. Nevertheless, despite NAC administration, mortality and morbidity rates in PARA toxicity remain high, prompting ongoing research for new and more effective therapeutic agents.^23^

ART is well-documented in the literature to possess broad pharmacological activities, including anticancer, anti-inflammatory, and antioxidant properties.^13^ Furthermore, Gilani et al. reported that the extract of *A. absinthium*, which contains the active compound ART, reduced ALT and AST levels in PARA-induced hepatotoxicity and confirmed its potential use in the traditional treatment of liver injury.^24^ Based on this information, it was hypothesized that ART could serve as a novel adjunctive agent to NAC in the treatment of acute PARA-induced liver toxicity. Therefore, in rats, biochemical liver function parameters such as serum ALT and AST levels, oxidative stress markers such as SOD, GSH, and MDA levels in liver tissue, molecular markers including TNF-α, IL-1β, iNOS, NF-κB, and CYP2E1 mRNA expression levels, and histopathological analyses were comparatively evaluated with NAC as the protective treatment.

First, when examining the serum levels of ALT and AST—important general indicators of liver injury—we observed surprising results in the PARA + ART 35 group when compared with the PARA group and the protective treatment groups. The ALT and AST levels in this group even exceeded those of the PARA group. In contrast, ALT and AST levels in the PARA + ART 7 and PARA + ART 14 groups decreased in a dose-dependent manner compared to the PARA group. In a study from the literature, it was demonstrated that in an alcohol-induced hepatotoxicity model, ART at doses of 60 and 120 mg/kg reduced ALT and AST levels in a dose-dependent manner and effectively prevented liver injury. The increase in therapeutic efficacy with higher doses supports our findings; however, the results obtained for the PARA + ART 35 group were found to differ from the data reported in the literature.^25^ In another study, different derivatives of ART at varying doses were administered to healthy mice, and while low doses did not alter ALT and AST levels, high doses were found to elevate these levels.^26^ Similarly, in another study, the effects of ART-based combination therapy on specific liver function indices were evaluated in healthy pregnant Wistar albino rats. The results showed that as the administered dose increased, serum ALT and AST levels also rose. These findings in the literature indicate that higher doses of ART may exert toxic effects.^27^

The primary mechanism underlying PARA-associated liver injury is the depletion of hepatic GSH stores. In this context, agents that increase GSH levels may protect against or reverse PARA-induced liver damage.^28^ Additionally, important indicators of tissue homeostasis include oxidative stress markers and antioxidant enzyme levels. In cases of PARA-induced hepatotoxicity, SOD activity is observed to decrease while MDA levels increase.^20^ In the present study, we observed that the decreased GSH levels and SOD activity in the PARA group increased in a dose-dependent manner in the PARA + ART 7 and PARA + ART 14 groups. However, in the PARA + ART 35 group, GSH levels and SOD activity were surprisingly lower than even those of the PARA group. The MDA parameter confirmed these findings: while the elevated MDA levels in the PARA group decreased dose-dependently in the PARA + ART 7 and PARA + ART 14 groups, the PARA + ART 35 group exhibited unexpectedly higher MDA levels compared to the PARA group. In a study by Farombi and colleagues, the effects of ART on sperm characteristics, antioxidant status, and hormonal profiles in rats were examined. ART was administered at 7 and 35 mg/kg, and it was reported that the 35 mg/kg dose decreased GSH levels in rat spermatozoa, whereas the 7 mg/kg dose did not. Both doses, however, increased SOD activity. Additionally, the 35 mg/kg dose elevated MDA levels in spermatozoa compared to the control group, while the 7 mg/kg dose showed no such effect. This study supports our findings regarding the dose-dependent effects observed on SOD activity in the PARA + ART 7 and PARA + ART 14 groups and corroborates the observations in GSH and MDA levels seen in the PARA + ART 35 group.^16^

Numerous studies have supported the antioxidant effects of ART. In models of doxorubicin-induced hepatic injury, administration of *Artemisia annua* extract containing the active ART compound led to upregulation of antioxidant gene expression.^29^ In another study investigating the effect of ART on total oxidant/antioxidant balance, ART was shown to reduce oxidative stress parameters in both the kidney and liver. These findings corroborate the results observed in our PARA + ART 7 and PARA + ART 14 groups.^30^

The selected mRNA markers (TNF-α, IL-1β, iNOS, NF-κB, and CYP2E1) were chosen based on their critical roles in the pathogenesis of paracetamol-induced hepatotoxicity. CYP2E1 is a key enzyme responsible for the bioactivation of paracetamol into the toxic metabolite NAPQI, which leads to oxidative stress. This oxidative stress subsequently activates NF-κB signaling, resulting in the upregulation of pro-inflammatory cytokines such as TNF-α and IL-1β, as well as iNOS. Therefore, these markers are mechanistically interconnected and collectively reflect oxidative stress, inflammatory response, and liver injury. The evaluation of these parameters provides a comprehensive understanding of the hepatoprotective effects of artemisinin.

In line with this mechanistic framework, inflammatory mediators have been shown to play a crucial role in PARA-induced toxicity.^31^ In addition to oxidant/antioxidant parameters, inflammatory mediators have also been found to be associated with PARA-induced toxicity. According to our TNF-α results, TNF-α mRNA expression levels in the PARA + ART 7 and PARA + ART 14 groups were decreased in a dose-dependent manner compared to the PARA group. Interestingly, the PARA + ART 35 group exhibited TNF-α levels exceeding those of the PARA group. When examining other inflammatory mediators, similar trends were observed, though not identical to TNF-α. Levels of IL-1β, iNOS, and NF-κB were significantly lower in the protective treatment groups compared to the PARA group, with the greatest reduction observed in the PARA + ART 14 group. No statistically significant difference was detected between the PARA + ART 7 and PARA + ART 14 groups, indicating that a clear dose-dependent effect could not be confirmed. In a study by Aktaş I. et al., TNF-α levels were elevated in the doxorubicin-induced toxicity group, while ART treatment resulted in a dose-dependent decrease in these levels.^15^ Furthermore, in studies on doxorubicin-induced testicular and renal injury, ART was reported to attenuate the overexpression of TNF-α, iNOS, NF-κB, and Caspase-3. These findings from the literature support and validate the results observed in our study.^32^

CYP2E1 is a key cytochrome P450 enzyme involved in ethanol metabolism and also participates in the metabolism of various xenobiotics, one of which is PARA.^33^ According to our results, CYP2E1 mRNA expression levels in the PARA + ART 7, PARA + ART 14, and PARA + ART 35 groups were reduced compared to the PARA group. However, the reduction observed in the PARA + ART 35 group was less pronounced than in the other groups, with the greatest decrease occurring in the PARA + ART 14 group. No statistically significant difference was detected between the PARA + ART 7 and PARA + ART 14 groups, indicating that a clear dose-dependent effect could not be established. In a study conducted in humans, the effect of ART on the cytochrome P450 family enzyme CYP1A2 was investigated, and administration of 500 mg ART was found to decrease CYP1A2 enzyme activity.^34^ Similarly, another study in rabbits demonstrated that ART reduced the activity of CYP3A4, another cytochrome P450 enzyme. Given that the parameter examined in our study is also a cytochrome P450 isoform, these findings are consistent with our results.^35^ Additionally, one derivative of ART, dihydroartemisinin, was reported to inhibit CYP2E1 activity when administered at doses of 25 and 50 mg/kg in mice with cisplatin-induced nephrotoxicity. This study is particularly relevant as it parallels our findings regarding the CYP2E1 enzyme.^36^

Finally, to assess liver injury at the cellular level, histological examinations and immunohistochemical staining were performed. While the CONTROL group displayed normal histology, marked cellular damage was observed in the PARA group. Interestingly, the PARA + ART 35 group also exhibited high levels of cellular damage, showing a pattern similar to the PARA group. In contrast, in the PARA + ART 7 and PARA + ART 14 groups, dose-dependent improvements in cellular integrity were evident. Immunohistochemical analyses corroborated the histopathological findings. TNF-α expression in the CONTROL group was minimal, with no noticeable immunoreactivity. In the PARA group, TNF-α expression was significantly elevated, exhibiting strong immunopositivity. Similarly, the PARA + ART 35 group showed a pattern resembling the PARA group rather than the protective treatment groups. However, in the protective treatment groups, namely PARA + ART 7 and PARA + ART 14, TNF-α expression decreased in a dose-dependent manner, with reduced immunoreactivity in hepatocytes and attenuated inflammatory responses. In a toxicity study conducted by Aktaş et al., the coadministration of ART with doxorubicin improved histopathological changes associated with doxorubicin toxicity, including hyperemia, edema, degeneration, and mild inflammatory reactions. In the same study, immunohistochemical staining was used to evaluate TNF-α and Caspase-3 expression in cardiac and hepatic cells. Regarding TNF-α, expression was observed only in myocardial cells and hepatocytes in the doxorubicin-only group, whereas no expression was detected in other groups. Examination of Caspase-3 expression in myocardial cells revealed a significant increase in the doxorubicin-treated group compared to the control group, while ART-treated groups showed a marked reduction in Caspase-3 expression relative to the doxorubicin-only group. In hepatocytes, Caspase-3 expression was present only in the doxorubicin-treated group, with no immunoreactivity observed in other groups. These literature findings support the results obtained in our study.^15^

In conclusion, this study demonstrated that ART, when administered at an appropriate dose, exerts hepatoprotective effects in PARA-induced liver injury, and these effects appear highly promising. We propose that ART may play a significant biological role in preventing PARA-induced hepatotoxicity by increasing GSH levels, reducing CYP2E1 enzyme expression, and acting through antioxidant and anticytokine mechanisms. However, certain aspects of our findings partially contrast with the existing literature, highlighting the need for more detailed investigations at the molecular level to elucidate ART’s hepatoprotective mechanisms and to evaluate its potential hepatotoxic effects across different dosage ranges. If our findings are corroborated by further studies, ART may represent a promising adjunctive agent alongside NAC in the management of PARA intoxication.

## Data Availability

Data will be provided upon a reasonable request.
